# WORK TO RETIREMENT: EXAMINING THE ROLE OF RETIREMENT IN THE ASSOCIATION BETWEEN AWARENESS OF AGING AND SLEEP HEALTH

**DOI:** 10.1093/geroni/igae098.2518

**Published:** 2024-12-31

**Authors:** Neha Dewansingh, Kristin Calfee, Claire Smith, Soomi Lee

**Affiliations:** University of South Florida, Davenport, Florida, United States; The Pennsylvania State University, University Park, Pennsylvania, United States; University of South Florida, Tampa, Florida, United States; The Pennsylvania State University, University Park, Pennsylvania, United States

## Abstract

Individuals of all ages require adequate sleep to maintain a healthy lifestyle; sleep health is especially vital in older adults, but often degraded after retirement. Poor sleep puts older adults at risk for physical and cognitive impairments, which can increase fall risk, difficulty performing daily activities, and chronic conditions like Type 2 diabetes. Awareness of aging (AoA), or consciousness around one’s aging experiences, has been linked to promoting health behaviors such as exercising. Sleep is a health behavior; yet its association with AoA has been little studied. Both sleep and AoA may change through retirement, and the association between them may also differ by retirement status. We investigated these relationships using a self-report composite measure of sleep health across multiple dimensions (i.e., regularity, satisfaction, alertness, efficiency, and duration) in a national sample of adults (n=668; Mage=63yrs) from the Midlife in the United States study, controlling for sociodemographic covariates and self-rated health. Results demonstrated that higher AoA was associated with worse sleep health for both retired and non-retired adults (B=-0.256, SE=0.083, p=0.002), with more pronounced association for retirees (B=-0.418, SE=0.178, p=0.019). Such findings suggest that higher awareness around aging surprisingly relates to greater disruptions in sleep health for older adults, particularly in retirees, counter to prior work emphasizing the health benefits of AoA. Future research could examine how and why retirees experience unanticipated negative side effects of AoA such as sleep disruptions. Limitations and directions for future research will be discussed, with potential methods for managing AoA by specific contexts.

